# Obstacles to implementation of an intervention to improve surgical services in an Ethiopian hospital: a qualitative study of an international health partnership project

**DOI:** 10.1186/s12913-016-1639-4

**Published:** 2016-08-17

**Authors:** Emma-Louise Aveling, Desalegn Tegabu Zegeye, Michael Silverman

**Affiliations:** 1 Cambridge Centre for Health Services Research, University of Cambridge, Institute of Public Health, Forvie Site, Robinson Way, Cambridge, CB2 0SR UK; 2 Department of Health Policy and Management, Harvard T.H. Chan School of Public Health, Boston, USA; 3 Federal Ministry of Health, P.O. Box-1234, Addis Ababa, Ethiopia; 4 Department of Infection, Inflammation and Immunity, University of Leicester, University Rd, Leicester, LE1 7RH UK

**Keywords:** Quality improvement, Surgery, Patient safety, Partnership, Ethiopia

## Abstract

**Background:**

Access to safe surgical care represents a critical gap in healthcare delivery and development in many low- and middle-income countries, including Ethiopia. Quality improvement (QI) initiatives at hospital level may contribute to closing this gap. Many such quality improvement initiatives are carried out through international health partnerships. Better understanding of how to optimise quality improvement in low-income settings is needed, including through partnership-based approaches. Drawing on a process evaluation of an intervention to improve surgical services in an Ethiopian hospital, this paper offers lessons to help meet this need.

**Methods:**

We conducted a qualitative process evaluation of a quality improvement project which aimed to improve access to surgical services in an Ethiopian referral hospital through better management. Data was collected longitudinally and included: 66 in-depth interviews with surgical staff and project team members; observation (135 h) in the surgery department and of project meetings; project-related documentation. Thematic analysis, guided by theoretical constructs, focused on identifying obstacles to implementation.

**Results:**

The project largely failed to achieve its goals. Key barriers related to project design, partnership working and the implementation context, and included: confusion over project objectives and project and partner roles and responsibilities; logistical challenges concerning overseas visits; difficulties in communication; gaps between the time and authority team members had and that needed to implement and engage other staff; limited strategies for addressing adaptive—as opposed to technical—challenges; effects of hierarchy and resource scarcity on QI efforts. While many of the obstacles identified are common to diverse settings, our findings highlight ways in which some features of low-income country contexts amplify these common challenges.

**Conclusion:**

We identify lessons for optimising the design and planning of quality improvement interventions within such challenging healthcare contexts, with specific reference to international partnership-based approaches. These include: the need for a funded lead-in phase to clarify and agree goals, roles, mutual expectations and communication strategies; explicitly incorporating adaptive, as well as technical, solutions; transparent management of resources and opportunities; leadership which takes account of both formal *and informal* power structures; and articulating links between project goals and wider organisational interests.

**Electronic supplementary material:**

The online version of this article (doi:10.1186/s12913-016-1639-4) contains supplementary material, which is available to authorized users.

## Background

Access to safe surgical care represents a critical gap in health service delivery and development in many low- and middle-income countries [1, 2], including Ethiopia [3]. Increased infrastructure and human resources are essential to address such national deficits in surgical capacity [4], and the Ethiopian government has embarked on an ambitious programme of healthcare reform which aims to increase human resources and improve hospital infrastructure. In May 2016, the Federal Ministry of Health also launched the Saving Lives Through Safe Surgery initiative (SALTS), which aims to promote safe surgery throughout the country to alleviate the national burden of diseases. While necessary, increased resources at a national level are unlikely to be sufficient on their own, however. Research from low-income countries also highlights the need for improved management and organisation of resources within hospitals [5], indicating that quality improvement at this level also has an important role to play in enhancing access to safe care [6–8].

Improving healthcare quality and safety is notoriously difficult [9, 10], and the results of improvement interventions are often disappointing [11]. Analysis of the reasons that interventions fail to achieve the desired results is essential if the science of healthcare improvement is to move forward [12]. While much has been learnt about the determinants of successful quality improvement efforts, the vast majority of research and evaluation pertains to high-income country settings. Yet the role of context in influencing whether and *how* an intervention ‘works’ is significant [11]. There is a particular need, therefore, to develop understanding of how to optimise quality improvement efforts in low-income (LIC) country settings [6]. An essential component of this endeavour is the use of process evaluations that examine *how* an intervention is enacted and help to identify barriers and facilitators to implementation [9, 13, 14].

One prominent approach to quality improvement efforts in LICs involves international collaborations with partners from high-income countries. Such international partnerships form an increasingly prominent approach to tackling healthcare quality and safety in Ethiopia, as elsewhere in Sub-Saharan Africa [15, 16]. International partnership initiatives in the Ethiopian context include, for example, the Clinton Health Access Initiative (which works with the government on a range of programs to improve access to and quality of health services), the World Health Organization’s (WHO) African Partnerships for Patient Safety programme (which supports and facilitates learning across hospital-to-hospital partnerships), and numerous institutional health partnerships between hospitals and/or universities in Ethiopia and high-income countries such as the UK. While advocated as a potentially valuable component in efforts to strengthen health services [17], including surgical services [18], international partnerships can be challenging and their impact is often mixed. Partnerships frequently face challenges relating to divergences in language, interests, priorities, and access to resources and education; these can lead to difficulties ensuring equal stakeholder involvement, ownership and commitment and in ensuring mutual understanding, clarity of purpose and coordination of collaborative efforts [19–22]. In addition to the obstacles presented by challenging implementation contexts, such difficulties can lead to failure to establish partnerships (or their breakdown), and limited or patchy success in achieving some or all of the goals of a specific project (such as improvements in health research capacity or healthcare quality and safety) [21–24]. Many hospital-to-hospital partnerships lack the resources to carry out in-depth process evaluation of their improvement initiatives [25], curtailing opportunities for much-needed documentation and sharing of learning [13, 26].

In this paper we report on the findings of a qualitative process evaluation of a quality improvement project in an Ethiopian University Hospital (anonymized as Borodar University Hospital). The project was designed and implemented in partnership with a UK hospital (anonymized as Glennworth) through the pre-existing Borodar-Glennworth partnership - a partnership which had been active for over a decade. The project, anonymized as the Surgical Capacity Improvement (SCI) project, aimed to strengthen the organisation and management of surgical services in Borodar University Hospital, a regional tertiary care centre serving over five million residents. At the start of the project (2011), the hospital had 500 beds, and performed ~6000 major surgical procedures annually across four operating rooms (ORs). Like many facilities which provide surgical services in Ethiopia [4], the context was a challenging one characterized by limited (albeit increasing) human resources (particularly anaesthetists and nurses) and unreliable access to electricity, running water, essential medications and equipment.

The SCI project was funded by a UK-based trust which supports partnership-based healthcare projects, and was implemented over 18 months. The project’s overarching goal was to improve access to surgical services for patients in Borodar based on the rationale that significant improvements could be made through better management of existing (albeit limited) resources. It sought to achieve this goal by: establishing a multi-disciplinary OR management committee; training all theatre staff in management skills; reducing ‘down-time’ in the OR and increasing through-put; and establishing clinical record-keeping and audit as routine management tools.

By the end of the 18-month implementation period, many of the proposed activities had not been implemented and almost none of the project objectives had been met. We sought to explain why. Through a process evaluation which examined project implementation and the implementation context, we identify key factors that contributed to the disappointing results of the project. We discuss the lessons for future improvement interventions, particularly those involving international partnerships.

## Methods

An independent, qualitative process evaluation of the SCI project was conducted (October 2011-January 2014) as part of a larger ethnographic study of international partnerships and efforts to improve healthcare quality and safety. The process evaluation aimed to examine how the project was enacted, with a focus on: implementation processes (how and with what degree of fidelity planned inputs, activities and strategies were implemented); how project partners and participants engaged with the intervention; and context characteristics which influenced implementation [12, 14].

Evaluation data comprised interview, observation and documentary data relating to the SCI project. This included 66 semi-structured interviews carried out at three time points: 22 at the start of the SCI project, 30 one year into the SCI project and 14 after the project had finished (Table 1). These time-points enabled longitudinal study of project implementation, from initial launch and development of implementation plans, through the implementation period to reflections following the end of project funding. We interviewed three categories of participants: 1) Glennworth partners (health professionals) involved in the SCI project (nine); 2) Borodar partners directly involved in the SCI project (seven); 3) Borodar surgical staff, including surgeons, trainees, anaesthetists, nurses and cleaners (35). All original SCI Borodar committee members were interviewed at least twice; two of the Glennworth project leads were interviewed twice; five other Borodar OR staff were interviewed twice. Interviews were conducted by ELA or AN (see Acknowledgements) in English or Amharic, translated (where necessary) and transcribed verbatim. Interviews covered views of the SCI project (aims, activities, achievements and obstacles), of the partnership and - to facilitate exploration of the implementation context - of problems encountered in routine OR functioning (see Additional file 1). ELA and AN were independent researchers, not directly involved in running the SCI project; nonetheless ELA in particular may have been seen by some as associated with the project due to shared institutional connections with the UK project team. This may have influenced participants’ responses; measures to reflexively take account of this possibility were taken in the approach to interviews, triangulation with other data sources and during analysis.

**Table 1:** Number and role of interview participants

	Pre-project implementation	Project implementation phase	Post-project end
UK SCI project team	3	6	1
Borodar SCI project team	4	5	5
Borodar OR staff	15	19	8
*Anaesthetic staff*	*4*	*6*	*2*
*Surgeon & Obs-Gyn staff*	*6*	*8*	*5*
*OR nurses*	*5*	*6*	*5*
Other OR staff	4	4	1
Total	22	30	14
Grand total	66		

ELA and AN also conducted 135 h of observations in the operating rooms before the project started, during the project and after the project had ended. This allowed us to observe efforts to implement changes through the SCI project, as well as routine functioning of the OR in order to gain understanding of the implementation context. ELA observed two meetings of Glennworth partners concerning the SCI project; the second meeting took place after the project had finished and included discussion of what had ‘gone wrong’ during the SCI project. Brief notes were jotted down during observations, then typed up in full by ELA. We also collected documents relating to the SCI, including the project proposal, reports to the funder and visit reports. Triangulation between data sources allowed us to build a holistic picture of the project activities, the implementation context and the challenges encountered.

Given the outcomes of the project, our analysis focused specifically on identifying the barriers and challenges to implementation. Data was coded by author ELA, using Nvivo (QSR) software, and the analysis was discussed, reviewed and agreed on by all authors. Thematic analysis of the data drew on theoretical constructs relating to collaborative projects [27, 28] to examine the components of the improvement intervention and their influence on outcomes. Specifically, we directed our analysis towards examination of participants’ understandings of: the project itself (its objectives and the strategies and resources proposed/needed to achieve these); the formal and informal rules and mechanisms guiding project team and partner interactions and communication; the division of labour among partners and project team members; and the engagement of the wider OR community in the project. Recognising the importance of contextual influences, we also sought to characterise the ways in which the implementation context shaped and constrained these intervention components. In line with our theoretical framework, the next step was to identify what Engeström [27] calls ‘contradictions’ within the system, i.e., mismatches between components, between components and context or between the perspectives of different stakeholders on these components. Such contradictions have the potential to undermine collaboration and project implementation [28].

## Results

The overarching goal of the SCI project was to improve access to the OR in terms of increased through-put of cases, reduced delays and decreased closure of individual ORs due to lack of equipment or drugs. Table 2 summarises the SCI objectives, the activities proposed to facilitate meeting these objectives, and the achievements of the SCI as reported in interviews and project documentation (funding proposal, reports to funder).

**Table 2:** Summary of project objectives, proposed activities and reported achievements

Project objectives	Proposed activities	Achievements
Overarching project objective: To improve access to the operating theatres for both surgical and obstetric patients in Borodar, by increasing the number of procedures and decreasing delays and periods of closure	Audits to assess: -Number of acute and elective procedures -Delays for acute obstetric emergencies -Amount of time ORs closed due to lack of functioning equipment or drugs	-None of the proposed audits were completed. -6-week audit of cancellations completed but not seen by all partners/project team members -No anecdotal evidence or reported observation of any improvement in through-put, delays or periods of closure
To meet objectives and carry out activites through on-going collaboration between Borodar and Glennworth partners	-Ongoing support and dialogue between partners via email -One visit by Borodar OR committee members to UK -Two visits by two Glennworth OR professionals to run training workshops and support the OR management committee	-Efforts to maintain dialogue between partners enacted, but significant challenges and misunderstandings reported -Two visits by Borodar OR committee members to UK were undertaken, though later than scheduled -One visit by Glennworth OR professionals to Borodar, though later than scheduled and training workshops not held.
To improve OR management through establishment of a functional, multi-disciplinary OR management team	-Identify and establish OR management team to include representatives of surgery, obstetrics, anaesthesia and nursing -Hold regular, minuted meetings.	-OR management committee members identified -Only two meetings held during 18-month project period
To train all OR professionals in OR leadership and management skills	-Run four two-day workshops on leadership and management for all qualified staff using the operating theatres and recovery (about 60).	-Proposed workshops on leadership and management not held -Senior OR nurse provided some training in waste management to nurses
To establish clinical record keeping and clinical audit in the operating theatres as routine management tools, and to monitor patient intra-operative morbidity and mortality	-Establish a reporting system to monitor adverse incidents, peri-operative morbidity (including infection rates) and mortality. -Establish a no-blame peri-operative morbidity and mortality review panel. -Establish 6-monthly clinical audit meetings -Create and use appropriate clinical records, including drug charts. -At least 1 completed audit in each 6-month period by each of the 4 professional groups in operating theatres and recovery.	-Reporting system not established -Morbidity and mortality review panel not established -6-monthly clinical audit meetings not held -New clinical records not created -1 audit of cancelled procedures completed by one of the professional groups; no other audits reported to be completed by other groups for the project (although some Masters students working in the OR completed audits for course requirements, separate to the SCI initiative)

Despite a great deal of initial enthusiasm for the SCI project from both teams and Borodar hospital management, and the dedication of significant time and effort from all team members, all partners agreed that the positive impacts on OR management they hoped for had not materialised. Most objectives had not been met and many activities were not implemented. An initial ‘launch’ meeting between Glennworth team leads and Borodar team leads was held (in Glennworth) to review the project proposal and plan initial steps. Following this meeting, the Borodar team leads (the hospital CEO and clinical director) identified four ‘frontline’ members (a nurse, an anaesthetist, a surgical resident and an obstetrics resident) to work alongside them to form the OR improvement committee. However, the committee only held two meetings during the 18-month project period. Two further visits were undertaken (one by two OR management committee members to Glennworth, the other by two Glennworth OR professionals to Borodar), but the proposed leadership and management training workshops for OR staff did not take place. The nurse representative on the committee did, though, provide training for OR nurses in waste management processes following his visit to the UK. Apart from a 6-week audit of cancelled procedures, routine clinical audits were not established, nor a review panel or regular OR audit meetings. There was no evidence (anecdotal or audit-derived) that the goal of improving access to surgical care for patients (increased OR through-put or reduced ‘down-time’) had been achieved.

To explain these outcomes, we present our findings in terms of three, interrelated sets of factors pertaining to: 1) the project design, 2) partnership working and 3) the implementation context in Borodar.

### Project design: objectives, resources and strategies

In terms of the QI project itself, we identified obstacles relating to (mis)understandings of the project objectives within the SCI team and to mismatches between the proposed resources, activities and aims.

#### Understanding of the project objectives

Despite collaborative planning by team leads, as the project progressed confusion emerged within the SCI team over the objectives of the project. While there was broad agreement that the aim was to improve the functioning of the OR, different team members had different views about what activities should be implemented to achieve this. Particularly towards the end of the project, team members tended to give a list of objectives (e.g., reducing clutter, instigating use of digital record keeping in the OR, improving waste management), which differed from one member to another, and most of which did not feature in the original proposal.

*I think initially we thought it was more about, sort of, morbidity, mortality and improving any rates that there were, but it turned out to be more about more, sort of, basic and ground floor things [..]So I don’t know that it’s exactly what we set out to do (project team member, Glennworth)*

These divergences resulted in different team members focusing on separate activities (e.g., training on waste management for nurses but not other staff, installing computers for use by surgeons) at the expense of coordinating time and efforts towards agreed project objectives. More seriously for the partnership relationship, disagreement over key activities – such as the number and direction of overseas visits - resulted in conflict and frustration between partners.

*I was disappointed that I was not involved in deciding on some of the things, and the other thing is that [the planned visit] was also not what I wanted to do (project team member, Borodar)*

#### Mismatched resources, aims and activities

Mismatches between the resources provided by the project and those needed to accomplish its aims and activities were another obstacle to success. The principal resource deficiency was staff time – this being a volunteer-run project. For example, Borodar team members felt hugely overburdened by existing clinical demands, and struggled to make time for meetings or implementing activities. Moreover, they were frequently called away by higher authorities at the last moment, disrupting planned activities. Financial pressures in the UK hospital meant that Glennworth staff found it more difficult than in the past to obtain permission for annual leave for project visits to Ethiopia, causing delays to the planned visits.

Establishing clinical audit was another key strategy of the project. Most OR staff had limited training in clinical audit. Although Glennworth partners were able to provide some technical support to some individual team members (through visits to the UK and email), the planned training for other OR staff did not take place. In addition, the OR committee did not include the hospital’s audit clerk, who had previously undertaken certified training through the partnership. An opportunity was missed therefore to utilise this individual’s skills and the dedicated time s/he had available to support audit within the hospital.

A further challenge was that many OR staff members tended to view audit as a potentially punitive tool which could lead to individuals being blamed, rather than as a tool for learning. For example, one senior physician described being angrily accused of ‘making trouble’ when he had shared the results of an audit with senior management in an effort to raise concerns about quality of care. As a result, individuals who did attempt to carry out audits in the OR sometimes encountered significant push-back from their colleagues. The project design included little support for overcoming these ‘adaptive’ challenges, instead focusing planned activities on addressing technical, skills-based needs.

### Partnership working

Significant challenges were encountered in maintaining effective communication, understanding and coordination of roles and responsibilities between partners.

#### Exchange visits – logistical challenges

Exchange visits between Ethiopia and the UK - a key activity to facilitate collaborative implementation - proved particularly problematic. Factors outside the control of the project team, such as difficulties obtaining visas for visitors from Ethiopia and personal illness, delayed visits and the second planned visit by Glennworth partners to Borodar did not happen. This caused serious delays to project progress and prevented planned training for OR staff from taking place.

*It seemed really likely to succeed because there was enthusiasm on both sides, but it hasn’t succeeded because there are a lot of external factors [..] the changes in the NHS meant there were delays in visits, changes meaning that visas are no longer reliably obtained by people from Ethiopia (project team member, Glennworth)*

#### Communication

Between visits, email communication between SCI project partners was poor, with emails going unanswered, or responses too slow or with too little information to allow project activities to move forward. Partners in the UK and Ethiopia blamed demanding clinical workloads, as well as a lack of clarity about who was supposed to send or answer project-related emails. In Ethiopia these issues were compounded by limitations in IT resources. The result was lack of coordination between team members and uncertainty on both sides about what was happening or what was planned. Over time, these communication failures stymied action and contributed to a loss of motivation on both sides of the partnership.

*There is some communication failure also between us and [Glennworth partners],[..] sometimes decisions will come from the other side and I really don’t know anything about it[..] so that is also another big problem that I observed (project team member, Borodar)*

*There was a great delay on both sides, but mainly on our side actually, on communications, so a month would go by and it doesn’t seem like very much, but then, you know, time, it just accumulates and it’s very frustrating for people waiting for a response. (project team member, Glennworth)*

Communication challenges appeared to have been compounded by differences in partners’ norms and expectations about appropriate interactions. One prominent example of this was how partners chose to communicate when the project was not progressing according to plan. On the Glennworth side, partners wanted to be informed even if the report was not positive; in their view, maintaining communication was the most important indicator of commitment to the project. Borodar staff suggested that according to local norms there was a reluctance to share bad news, such that avoiding disappointing partners was prioritised over sharing updates.

#### Project team roles, responsibilities and mutual understanding

Communication was also hampered by a more general lack of clarity or mutual understanding amongst partners about project roles and responsibilities, particularly regarding project leadership. There was confusion between partners and within each partnership team about who should have oversight of project progress and who had the authority to finalise decisions and instigate action.

*I said to [another team member], you’re in charge of this bit, you and [another person] are the people who are leading it, but they wanted me to do it and I didn’t want to (project team member, Glennworth)*

This confusion was underpinned by a more fundamental mismatch in partners’ expectations. While Borodar team members expected Glennworth partners to take the lead and provide direction (e.g., suggest plans for addressing the problems identified), Glennworth members expected the Borodar team to take the lead and saw their own role as providing assistance in response to specific requests.

*We didn’t do anything, because I thought they would come and give us some directions and things like that, that was my thinking (project team member, Borodar)*

*It was discussed that we would go out there, but we were really keen they should come here first [..] I think it needs to be led by them. (project team member, Glennworth)*

Misunderstandings also emerged over the scope of the role of UK partners in relation to service improvement locally. For example, tension arose between partners when the application of hospital policy – rotation of nurses between different departments – meant that a key member of the Borodar project team left the OR. From the perspective of Glennworth partners, this move undermined the goal of strengthening the management of the OR, calling the appropriateness of the policy itself into question. From the perspective of the Borodar partners, such hospital-wide policies, which aimed to address wider challenges in hospital management, were felt to be beyond the scope of the project and therefore outside the scope of appropriate intervention from their partners.

Lack of clarity over roles and responsibilities was further complicated due to changes in personnel during the project (reflecting a wider issue of high staff turnover): by the end of the project, three of the five OR committee members were no longer in their original posts and able to work on the project.

### Local implementation context

The implementation context in Borodar presented significant challenges for establishing multi-disciplinary teamworking and for engaging the wider OR staff in improvement efforts.

#### Inter-disciplinary teamwork

The local context was characterised by a tendency for different professions (nursing, anaesthesia, surgery) to work in silos, with very few extant, formal structures to support inter-disciplinary collaboration. At times relationships between the different professions in the OR were strained, and conflicts were not uncommon.

*They [surgeons] say, “with how many people should we have to fight? We fight with anaesthesia, then should we also have to fight with the nurses?” (OR nurse)*

Given this context, the ‘adaptive’ work [29] – targeting culture and systems – of establishing a robustly functioning inter-disciplinary management committee was perhaps underestimated in the project design. While relationships between the individuals on the committee were cooperative, the local context made it difficult to coordinate project activities across the disciplines. Competing demands from within their own disciplinary departments made it hard for committee members to schedule group meetings. In addition, the relatively junior position of the surgery and obstetrics-gynaecology representatives (both residents) limited their influence within their own departments, and their efforts tended to be viewed by colleagues as their personal project, rather than a collectively-owned departmental activity.

#### Engaging OR staff: hierarchy, resource scarcity and structural constraints

The OR committee included representatives of each of the frontline professions working in the OR – surgery, obstetrics-gynaecology, anaesthetics, nursing – as well as the two hospital senior managers. All the frontline OR committee members lacked seniority, however. The obstetrics and surgical representatives were residents and although the nursing representative was the senior OR nurse, he lacked authority over non-nursing OR staff. In a steeply hierarchical context, this lack of authority proved very limiting to their efforts to secure compliance from senior surgeons in particular.

*The relationship between workers here is the relationship of boss and servant. The relationship is mostly based on inferiority and superiority. No sprit of team work at all. (OR nurse)*

*He [senior nurse] needs help from the physician’s side, but the doctors are a problem even for us, definitely there is, especially the senior doctors, they don’t cooperate. (project team member, Borodar)*

Many OR staff were unaware of the SCI project or its aims, as were UK doctors working in the OR on other partnership projects. OR staff who were aware of the project tended to view it as the project of one or two individuals, and any activities as their sole responsibility, indicating a lack of shared ownership amongst the wider OR staff.

*Most of the things probably the surgeons don’t know about, I don’t know about this project [..] they did appoint some people, the improvement committee, […] they can discuss the problems and find a solution, but all those things are the responsibility of this OR improvement committee. (surgeon)*

Although many OR staff agreed that resources could be better managed (e.g., many complained that time and resources were wasted due to poor organisation), all those interviewed saw *increasing* resources as the “burning issue” they wanted addressed. This is somewhat contradictory to the assumption underpinning the project design that deficiencies in the functioning of the OR were “not due to lack of staff or skill or undue financial pressure ….but due to the deployment of these resources” (project proposal). As a result, those staff who were aware of the project were skeptical or indifferent to efforts to introduce changes in practices or policies in so far as they thought the project was not tackling the main problem they faced.

*We don’t have simple gloves […] So how can people be happy to work? Whatever new policy, they don’t accept [..] unless you correct such very minor problems, how can you correct the big problems, how can you make people change? (surgeon)*

Overseas visits also appeared to be a source of tension between project staff and other Borodar OR staff. The opportunity for learning and professional development offered by visits to hospitals in countries such as the UK was rare but highly sought after. Some SCI team members suggested other OR staff felt decisions about who visited the UK partner hospital were unfair or inappropriate, and reported hearing rumours that staff who visited the UK through the SCI project had received personal, financial gain from doing so. Such rumours were likely exacerbated by a concurrent WHO project which had provided high daily allowances to staff who had undertaken WHO-supported visits to the UK. While SCI team members had not received daily allowances during visits, they nonetheless felt that such misunderstandings contributed to the resistance they encountered from OR colleagues in their efforts to implement project activities.

The hospital managers involved in the project were the most senior managers in the hospital. Due to the duties of this role in addition to clinical work, and the tendency for senior leaders to be called away from the hospital by regional or national authorities with little warning, senior leaders found it hard to actively participate in project activities, despite expressing much support for its importance. They also felt their ability to control or influence healthcare workers was limited.

*I tried, I struggled so many times to establish an OR policy, but it was not an easy thing, because people were not cooperative (senior manager, project team member, Borodar)*

Many of the reasons for this relate to the wider context. For example, the scarcity of human resources made it more difficult for managers to control doctors who, they felt, could easily move to other jobs. Low salaries, combined with demanding workloads in challenging material conditions were felt to contribute to low morale and motivation amongst healthcare workers, while also making it difficult for managers to identify incentives to help secure compliance.

*You know, they are here because they don’t have any other choice, the amount of money that they get is very small and they are not really well motivated […]we don’t do anything from the higher office, because if you do it’s going to affect them so much (senior manager)*

In this particular context, there were additional structural constraints that limited the influence of hospital managers over surgeons. In Ethiopia, most teaching hospitals fall under the jurisdiction of the Ministry of Education and are managed through their associated university. As a result, surgeons were employed by and accountable to the University whose first priority was academic rather than clinical. Thus hospital managers could not, for example, mandate that surgeons adhered to any of the new processes the improvement committee attempted to introduce.

Another structural obstacle to project progress and sustained engagement of the wider OR community was high staff turnover in the OR, particularly amongst nurses and trainees. This limited the beneficial impact of the training that was conducted, and made it more difficult for the project team to maintain awareness of and support for the project amongst OR staff.

*The OR, is a very very, hardworking place, and you have to work the whole day, the whole night, and then the next day, so [nurses] get exhausted, so after some time, they say “I’m leaving” (surgeon)*

## Discussion

The SCI project aimed to strengthen the organisation and management of surgical services in Borodar University Hospital through establishing a multi-disciplinary management committee, training OR staff and establishing clinical audit as a routine management tool. Despite the efforts of an enthusiastic and dedicated group of individuals, and broad agreement that improved management of the OR was much needed, the SCI project largely failed to achieve its goals. The context of intervention was a challenging one of resource constraints, high staff turnover, poor inter-disciplinary coordination and steep authority gradients within and between professions in the OR. These challenges are not unique to this hospital, nor the Ethiopian healthcare system [2, 7, 30]. In discussing the findings of this process evaluation, our intention is not to focus on resolving the contextual constraints per se, but to generate lessons for optimising quality improvement interventions within such challenging healthcare contexts, with specific reference to international partnership-based approaches.

### Strengthening project design and partnership working

A major problem for the SCI project were disagreements and ‘drift’ concerning project goals and strategies, and confusion over project roles and responsibilities. High staff turnover and delays caused by factors beyond the control of the project team undoubtedly contributed to the confusion and drift over time, but our findings also point to lessons for improving project design, collaborative planning and mutual understanding between partners.

Communication difficulties were significant, and affected implementation in practical ways (e.g., undermining coordination, delaying action), as well as negatively impacting on the relationships and motivation of team members. These were critical resources given the reliance on volunteerism and the challenges of finding the time for project work given existing pressures on clinical staff. Plans for communication (as well as implementation activities) need to be part of project design. Explicit agreement should be reached at the outset of a project about expected frequency of project updates or ‘virtual’ meetings, preferred mode(s) of communication and who has the authority to speak on behalf of others. It should include plans for periodically reviewing goals and strategies and renewing consensus as a team so that new learning can be incorporated without undermining coordination. Given local IT limitations, a small budget to support the costs of accessing reliable communication platforms may be needed; expanding the range of potential communication platforms may also be beneficial (e.g., Skype, WhatsApp, Dropbox etc.) [31]. Communication amongst team members on the same arm of the partnership should not be obscured by the focus on communication challenges between partners, however: our findings show that misunderstandings and confusion were also due to lack of dialogue between team members in the same hospital.

Our findings also point to more fundamental challenges for dialogue and mutual understanding, relating to mismatched expectations between partners about appropriate dialogue and sharing of information. Although the Glennworth-Borodar partnership was long standing, with many successful collaborations in the past, our findings suggest that longevity is not sufficient to ensure mutual understanding of partners’ roles and appropriate forms of interaction between the individuals involved in a specific project (some for the first time). This is perhaps not surprising, as smooth collaboration within partnerships is a common challenge in all fields [20]. The beliefs and assumptions underpinning expectations of partnership roles, as well as project-specific roles, need to be made explicit, and differences or contradictions resolved. This should include explicit consideration and agreement on the scope – and boundaries – of the proposed, collaborative intervention. While short-term QI projects clearly should be aligned with wider organisational goals, and may contribute to their development, our findings highlight the tensions for international partners raised by the interdependence of contexts and projects. In the longer-term, there is a need to address the hospital and system-level contextual constraints that can undermine specific QI efforts. But difficult questions arose about the extent to which wider hospital policies or systems were considered a legitimate ‘target’ for the project and the involvement of international partners. In challenging contexts such as this one, the boundaries of short-term, narrowly focused QI projects may be inherently ‘fuzzy’ and particularly complex for partnership-based approaches to negotiate.

Establishing teams, relationships and mutual understanding of the objectives and scope of a project necessarily takes time, but one approach to facilitating mutual understanding is to develop a concordat for each project undertaken by a partnership [32]. This should include agreement not only on what should be done, but how, making explicit the skills and support needed and/or expected. It should be a participatory process involving all team members, as the process of surfacing and discussing expectations is likely to be as valuable to sustaining good collaborative relations as the output itself (the concordat) [32]. Such a process takes time, and, in the context of an international project may be difficult to facilitate before funding is secured. An important lesson from our findings, then, is the need for a ‘lead-in phase’, *once funding is secured*, to allow greater attention to intervention design and planning with the involvement of all those to be directly engaged in implementation. Funding cycles may also need to be extended to allow time for teams, relationships and mutual agreements to be established, as short funding cycles of 12 to 18 months may make it hard to resist a rush to implementation [9].

### Tailoring implementation to context in resource-constrained settings

Another key area of difficulty for the SCI project was securing buy-in and support from other OR staff. The challenge of convincing staff that the problem being targeted is indeed a problem, or that it is the ‘right’ problem to target, is by no means unique to LIC contexts [9]. Our findings do suggest, though, that some features of LIC contexts may amplify these common challenges. First, resource constraints affected healthcare *improvement* efforts in particular ways. Lack of engagement stemmed in part from mismatches in the rationale of the project (that improvements could be made within existing resources) and the dominant perception that the ‘burning issue’ that needed addressing was the need to increase resources. In a context of stark material deprivation, it may arguably be harder to demonstrate the relative advantage of an intervention that did not directly seek to increase resources. Drawing on an observational study of a successful QI project in Rwanda, Kotogal and colleagues (2009) conclude that while QI tools can improve resource management, in resource-poor settings sustained change also requires *augmenting* resources [7]. The potential for the allocation of project-related resources and opportunities (e.g., for overseas visits or training), to engender resentment and resistance also presented challenges. As other successful projects have found, extrinsic motivators can be a valuable addition but require transparency and sensitive handling [33].

Second, a shorter national and local history of quality improvement and a low-base of staff morale meant that the scale of the *adaptive* challenges related to developing systems and a culture supportive of improvement efforts were particularly acute. For example, introducing clinical audit as a routine management tool required technical solutions, such as developing tools and training individuals. Some of this was provided, although obstacles outside the project team’s control prevented planned training workshops from being carried out. However, establishing routine audit also encountered sociocultural obstacles, namely a tendency to see audit as a potentially punitive tool (rather than a tool for improvement) and consequently resistance to its routine use. Similarly, establishing multi-disciplinary management of the OR was not simply a technical or practical task, but one which required adaptive solutions [29] to overcome structural inhibitors and deeply engrained professional norms that ran counter to multidisciplinary cooperation and collaborative management.

It is especially important, then, that QI projects in LICs include explicit strategies to secure buy-in and to overcome the adaptive challenges as well as the (perhaps more easily identifiable) technical challenges. In the SCI project, rather than allow the logic of the intervention to speak for itself, more needed to be done to communicate the rationale behind the proposed changes to OR management and practices. Opportunities were needed (e.g., at routine staff meetings) for discussion and the airing of objections as part of process of engagement [34], and to establish the collective benefit of the project and avoid the perception that the project ‘belonged’ to a few individuals.

For projects such as the SCI, largely reliant on volunteerism and persuasion in a context of relatively low staff morale, seeing results may help motivate enthusiasm and sustain commitment to improvement efforts [7, 33]. Another strategy to enhance engagement may be to identify and target low-hanging fruit, and so allow for ‘small-wins’ to be demonstrated early on, rather than aim only for ambitious, longer-term goals [9, 35].

Overseas visits were a particularly contentious aspect of the project – a source of delay due to logistical difficulties, of disagreement within the project team and of resentment from other OR staff members. Visits to the UK were highly valued in that they allowed for face-to-face discussions, the opportunity to learn and generate new ideas, and, for those making visits, were highly motivating. However, it was unclear that this value offset the delays and tensions they also engendered. An alternative may be to identify high-performing hospitals within country (or the region) and for international partners to visit those hospitals together. This would allow learning from other, high-performing sites, decrease logistical obstacles and funds spent on overseas travel, and counter resistance based on objections that what is done in the UK is inapplicable locally.

The importance of adaptive solutions and securing buy-in also underscores the need for leaders with the professional status, seniority or organisational position to influence decision making and the conduct of their colleagues [5, 7, 36]. While hierarchy is feature of healthcare and surgery globally, this differential is often more marked LIC settings [5]. Fragmented lines of authority within the institution, and the gap between the authority that team members’ professional or organisational position afforded them and that needed to persuade other OR staff to support the improvement efforts, indicate involvement of departmental heads and university (not just hospital) leadership would have benefitted the SCI project. Project leadership needs to reflect the specific, local accountability structures and include those at the highest level [31].

It is important to recognise, though, that in any setting power dynamics often extend beyond formal structures of authority and accountability [37]. As others have found [36], managers in our setting reported feeling they had little control over staff and few ‘hard edges’ at their disposal. It may help leaders, therefore, to articulate more clearly how short-term projects align with existing interests and the wider ‘direction of travel’ of the organisation (e.g., in this case, the links between the goals of the SCI and the Ethiopian Hospital Reform Implementation Guidelines). Moreover, as Bradley et al. [16] argue, management is not simply based on technical expertise but a process learned over time, and thus strengthening management may require on-going face to face mentoring in addition to short-term, didactic technical training.

## Conclusions

Through a process evaluation of an intervention which aimed to improve the management of surgical services, and identifying the barriers to implementation, we have sought to generate lessons for optimising quality improvement interventions in LICs. Although based on a single case, we have identified barriers which are common to improvement interventions in diverse settings, as well as obstacles relating to contextual features which are common to many LIC settings. We have also identified challenges relating to international partnership-based approaches specifically. We therefore hope the lessons generated may be useful for others seeking to conduct healthcare quality improvement in LICs, including through international partnerships.

Key lessons include:

- □ The need for a funded lead-in phase to enable clarification and agreement on roles, responsibilities and the skills, support and resources needed by those charged with implementation; this should be participatory, and include plans for sustaining effective communication both within and between partnership teams;
- □ The need to explicitly incorporate adaptive, as well as technical, solutions, and strategies for engaging those not directly involved in the project; this includes allowing space for debate and challenge of the project goals and rationale, avoiding excess ambition and allowing for early, small wins;
- □ The importance of careful and transparent management of the allocation of resources and opportunities for professional development, and realistic evaluation of the additional resources needed to address technical and adaptive challenges;
- □ The need to identify and engage local leaders whose professional status and organisational position afford them authority and influence given local formal *and informal* structures of power and accountability;
- □ The importance of identifying and articulating links between project goals and wider organisational interests and priorities, while also recognising and agreeing the scope of the QI project.

Developing the relationships and mutual understanding to underpin collaborative work, and laying the groundwork to create alignment, engage staff and promote local ownership takes time, especially where geographical and cultural distances need be crossed. An important lesson for funders, then, is to avoid funding cycles that are too short to avert an unhelpful, if well-intentioned, rush to implementation.

## Additional file

Additional file 1: Topic guides. (PDF 331 kb)

## Abbreviations

LIC: Low-Income Country
QI: Quality Improvement
SALTS: Saving Lives Through Safe Surgery
SCI: Surgical Capacity Improvement Project
WHO: World Health Organization
