# Supplementary material for: Obstacles to implementation of an intervention to improve surgical services in an Ethiopian hospital: a qualitative study of an international health partnership project
Source: BMC Health Serv Res. 2016 Aug 17;16:393. doi: 10.1186/s12913-016-1639-4 (PMC4987978; doi:10.1186/s12913-016-1639-4)
Supplement: Additional file 1: — Topic guides. (PDF 331 kb) [file 12913_2016_1639_MOESM1_ESM.pdf]

ADDITIONAL FILE 1: TOPIC GUIDES

A) Topic guide for Glennworth hospital staff involved in the partnership and SCI project

The partnership

- Can you tell me about your role/job in [Glennworth]?
- Can you tell me about how and why you got involved in the [Borodar-Glennworth] Partnership?
- What do you understand to be the aims of this partnership?
- What do you think working in partnership *should* mean?
- Has this partnership resulted in any benefits/ What are the potential benefits?
    - For you personally
    - For your hospital [Glennworth]
    - For staff in [Borodar]
    - For patients in [Borodar]
- What are/ were the main challenges of working in partnership?
    - How might these be addressed or overcome? / How have you tried to address these challenges?
- How could the partnership process/programme be improved?

Context

- Where in [Borodar] hospital did you visit/have you worked?
- What do you think are the main differences between [Borodar] and [Glennworth] hospitals?

Thinking about [Borodar] hospital:

- What do you think are the main strengths of the hospital?
- What do you think should be the main priorities for the hospital in terms of improving the quality and safety of services?
- What do you think are the strengths of the surgery department/OR?
- What do you think are main challenges faced by staff in the OR?
- What do you think are the main obstacles to providing access to quality surgical care?
- What, if any, features or aspects of the hospital or surgery department do you think facilitate efforts to improve quality and safety of surgical care?
- What, if any, features or aspects of the hospital or surgery department do you think impede efforts to improve quality and safety of surgical care?

The SCI project

- Can you tell me about how and why you got involved in the SCI project specifically?
- What do you know about how this project came about?
- How would you describe your role in this project?
    - How would you describe the role of your partners in this project?
- What do you understand to be the goals and aims of this project?
- What are the main strategies or activities through which you hope/d to achieve these aims?
    - What activities (inputs) are/ were planned?
- *Taking each of the activities/ strategies mentioned by participant*:
    - To what extent have you been successful in implementing/ completing this activity?
    - What are/ were the main successes?

    - What factors contributed to these successes?
    - What are/ were the main challenges?
    - How will/ did you try to tackles these challenges?
- *Prompts for activities:* Can you describe your experience to date of planning / implementing / supporting:
    - The activities of the OR management committee
    - Training workshops for OR staff
    - Activities relating to audit
    - Activities relating to clinical record keeping
    - Activities relating to M&M meetings
    - Activities relating to establishment of a reporting system
- How do you plan to / are you / did you manage the coordination and collaboration with your partners/team members in:
    - [Borodar]
    - [Glennworth]
- Overall, to what extent do you feel you have achieved the aims of the SCI project [to date]?
- What are/ were the main successes?
    - What do you think was important in allowing you to achieve these successes?
- What are/ were the main challenges?
    - And how did you overcome these?
    - What persisting challenges do you face?
- What are you most proud of?
- What would you do differently next time?

- Is there anything else you would like to add? Is there anything you think is relevant that we haven't covered?
- Do you have any questions?
- Thank you for your time.

B) Topic guide for Borodar hospital staff directly involved in the SCI project and Borodar-Glennworth partnership

Context
- Can you explain your job/role in this hospital?
- How would you describe this hospital – e.g. in relation to other hospitals in [location] or that you have worked in?
- What are the main strengths of this hospital?
- What are the main challenges for staff working in this hospital?
- What do you think are the strengths of the surgery department/OR?
- What do you think are main challenges faced by staff in the OR?
- What do you think should be the main priorities for this hospital in terms of improving the quality and safety of surgical services?

- What, if any, features or aspects of the hospital or surgery department do you think facilitate efforts to improve quality and safety of surgical care?
- What, if any, features or aspects of the hospital or surgery department do you think impede efforts to improve quality and safety of surgical care?

The partnership

- Can you tell me about how and why you got involved in the [Borodar-Glennworth] Partnership?
- What do you understand to be the aims of this partnership?
- What do you think working in partnership *should* mean?
- Has this partnership resulted in any benefits/ What are the potential benefits?
    - For you personally
    - For staff here
    - For patients here
    - For the UK partners
- What are/ were the main challenges of working in partnership?
    - How might these be addressed or overcome? / How have you tried to address these challenges?
- How could the partnership process/programme be improved?

The SCI project

- Can you tell me about how and why you got involved in the SCI project specifically?
- What do you know about how this project came about?
- How would you describe your role in this project?
    - How would you describe the role of your partners in this project?
- What do you understand to be the goals and aims of this project?
- What are the main strategies or activities through which you hope/d to achieve these aims?
    - What activities (inputs) are/ were planned?
- *Taking each of the activities/ strategies mentioned by participant*:
    - To what extent have you been successful in implementing/ completing this activity?
    - What are/ were the main successes?
    - What factors contributed to these successes?
    - What are/ were the main challenges?
    - How will/ did you try to tackles these challenges?
- *Prompts for activities:* Can you describe your experience to date of planning / implementing / supporting:
    - The activities of the OR management committee
    - Training workshops for OR staff
    - Activities relating to audit
    - Activities relating to clinical record keeping
    - Activities relating to M&M meetings
    - Activities relating to establishment of a reporting system
- How do you plan to / are you / did you manage the coordination and collaboration with your partners/team members in:
    - Borodar
    - Glennworth
- Overall, to what extent do you feel you have achieved the aims of the SCI project [to date]?

- What are/ were the main successes?
    - What do you think was important in allowing you to achieve these successes?
- What are/ were the main challenges?
    - And how did you overcome these?
    - What persisting challenges do you face?
- What are you most proud of?
- What would you do differently next time?

- Is there anything else you would like to add? Is there anything you think is relevant that we haven't covered?
- Do you have any questions?
- Thank you for your time.

C) Topic guide for Borodar hospital staff working in the surgery department

Context
- Can you explain your job/role in this hospital?
- How would you describe this hospital – e.g. in relation to other hospitals in [location] or that you have worked in?
- What are the main strengths of this hospital?
- What are the main challenges for staff working in this hospital?
- What do you think are the strengths of the surgery department/OR?
- What do you think are main challenges faced by staff in the OR?
- What are the main obstacles to securing safe care for surgical patients?
- What do you think should be the main priorities for this hospital in terms of improving the quality and safety of surgical services?
    - What changes would you most like to see?
- What, if any, features or aspects of the hospital or surgery department do you think facilitate efforts to improve quality and safety of surgical care?
- What, if any, features or aspects of the hospital or surgery department do you think impede efforts to improve quality and safety of surgical care?

The partnership
- Have you heard about or are you/have you been involved in the [Borodar-Glennworth] Partnership? How?
    - <u>If relevant:</u>
- What do you understand to be the aims of this partnership?
- What do you think working in partnership *should* mean?
- As far as you are aware, what kind of activities is/ has the partnership carried out here in [Borodar]?
- Has this partnership resulted in any benefits/ What are the potential benefits?
    - For you personally
    - For staff here

    - For patients here
    - For you’re the UK partners
- What are/ were the main challenges of working in partnership with international partners?

The SCI project

- Have you been involved in any surgical/ patient safety interventions in this hospital?
    - If so, which and in what way?
- Have you heard of the SCI project? / Have you been involved in this project in any way?
    - <u>If relevant:</u>
- Can you tell me about this initiative –
    - What are/ were its goals?
    - Who is/ was involved?
    - What activities or changes does/ did it involve?
- What kind of impact do you think this initiative is having/ has had in the department? (positive or negative)
- What do you think the main successes/ achievements of this project are/were?
- What factors contributed to any successes/ improvements in surgical care?
- What were the main challenges for or problems with this initiative?
- Are there other approaches to improving surgical care that you think may have worked more effectively?

- Is there anything else you would like to add? Is there anything you think is relevant that we haven’t covered?
- Do you have any questions?
- Thank you for your time.
